# Predicting parental caregiving burden based on sensory processing patterns and social skills in individuals with high-functioning autism spectrum disorder

**DOI:** 10.3389/fpsyt.2026.1747494

**Published:** 2026-03-05

**Authors:** Sale Zhang, Lili Zhang, Ying Li, Yu Zhang, Li Su

**Affiliations:** 1 Medical School, Xi’an Peihua University, Xi’an, Shaanxi, China; 2 Shaanxi Provincial Mental Health Center, Xi’an, Shaanxi, China; 3 Neurology Department, Xi’an Gaoxin Hospital, Xi’an, Shaanxi, China

**Keywords:** autism spectrum disorder, parental caregiving burden, prediction model, sensory processing, social skills

## Abstract

**Objective:**

This study aimed to Predicting Parental Caregiving Burden Based on Sensory Processing Patterns and Social Skills in Individuals with High-Functioning Autism Spectrum Disorder.

**Method:**

This correlational study recruited a convenience sample of 60 autistic children (aged 5–12 years, IQ > 80) from rehabilitation clinics. Parents completed the Zarit Burden Interview, the Sensory Profile School Companion, and the Gresham and Elliott Social Skills Rating Scale. Data were assessed using Pearson correlation as well as multiple regression analyses.

**Results:**

Sensory processing patterns were positively correlated with parental burden (r = 0.52 to 0.58), while social skills were negatively correlated (r = -0.59). Regression analyses identified significant unique predictors. Sensory Sensitivity (β = 0.28, p = 0.03) and Sensory Avoiding (β = 0.31, p = 0.02) were significant positive predictors of burden. In a separate model, lower Social Skills (β = -0.42, p = 0.003) and higher Problem Behavior (β = 0.28, p = 0.02) significantly predicted greater burden, collectively explaining 41.4% of the variance.

**Conclusion:**

Specific sensory processing difficulties (sensitivity and avoidance) and social-behavioral factors are significant, unique predictors of parental care burden. Interventions targeting these domains may alleviate caregiver stress and improve family outcomes in autism.

## Introduction

Autism Spectrum Disorder (ASD) is classified in the DSM-5 as a neurodevelopmental disorder characterized by persistent deficits in social communication and interaction deficits, together with restricted and repetitive behaviors, interests, or activities (1). The diagnostic criteria encompass impairments in social/emotional reciprocity, nonverbal communication behaviors, and the development and maintenance of relationships, combined with stereotyped behaviors and inflexible routines (2). Although clinical profiles vary, core deficits are consistently observed across domains of social engagement, communication, and repetitive behaviors (3).

The DSM-5 does not use the diagnostic specifier “high-functioning autism.” Instead, it classifies severity based on levels of support required for social communication and restricted, repetitive behaviors. For clarity in describing our specific sample, we use the term *autistic children with low support needs* operationally to refer to children who received a clinical ASD diagnosis, have a full-scale IQ > 80, and were reported by clinicians and caregivers to require minimal-to-moderate support (Level 1) in the aforementioned domains, despite experiencing significant challenges in social and sensory functioning (4). This operational definition is intended to precisely characterize the participants in this study.

Regarding language, this manuscript intentionally employs identity-first language (e.g., “autistic children”), as this reflects the expressed preference of many within the autistic self-advocacy community, who view autism as an integral aspect of identity. We use this terminology consistently to align with this perspective and to acknowledge the diversity of language preferences in the field, moving away from interchangeable use with person-first language.

Studies indicate that the caregiving and psychological burden associated with raising an autistic child profoundly impacts caregivers, presenting significant familial challenges (5). This burden manifests in objective dimensions—such as disrupted family dynamics, social constraints, and economic strain—and subjective dimensions, including internalized psychological reactions like depression, anxiety, and stigma (6).

Research demonstrates that for mothers, often the primary caregivers, having an autistic child is a chronic stressor adversely affecting mental health and adjustment, with fatigue being a prevalent outcome (7). Children’s behavioral challenges can lead to parental exhaustion, which may precipitate less effective coping strategies and heightened stress. This persistent pressure can erode responsive parenting, potentially creating a cycle where child behaviors trigger frustrated or punitive responses (8).

Furthermore, social skill deficits are a core feature of ASD. These include impairments in verbal and non-verbal social communication, difficulties in developing peer relationships, and absent social-emotional reciprocity (9). While these skills emerge organically in neurotypical development, autistic children exhibit marked deficiencies, which are crucial for academic success and long-term psychosocial adjustment (10). Evidence indicates that children with disabilities experience lower social adjustment.

These social skill deficits directly heighten parental caregiving burden (11). Autistic children may resist normative physical contact and struggle to recognize and express emotions conventionally (12). Even those with low support needs face core difficulties in establishing friendships, cooperating in groups, and demonstrating contextual empathy (13). Parent-mediated social skills interventions can significantly improve child behaviors (14, 15), alleviating parental burden by enhancing child flexibility and adaptation (11), with gains showing stability over time (16).

Such interventions can create a positive feedback loop, improving family interactions and reducing care-related tensions. These improvements foster more positive parent-child engagement, creating a more supportive family environment that benefits both child quality of life and parental mental health (17).

Beyond social-communication challenges, autistic individuals often experience co-occurring sensory processing differences (18), which predict social skill levels (19). Sensory processing involves the neurological registration, modulation, and integration of sensory information to produce adaptive responses, enabling effective environmental interaction (20). In autistic children, these differences may manifest as tactile defensiveness, sensory hypersensitivity or seeking, poor motor coordination, and related anxiety or avoidance behaviors (21), making daily participation challenging.

Despite the clinical significance of sensory and social factors, few studies have concurrently investigated their combined role in predicting care burden for parents of autistic children with low support needs. This gap is salient, as children’s often perplexing behaviors can significantly amplify caregiving pressures. Thus, this study aims to address: To what extent can sensory processing patterns and social skills predict the care burden experienced by parents of autistic children operationally defined as having low support needs?

## Methods

### Design and participants

The present study employed a descriptive-correlational design. The target population consisted of autistic children with low support needs, aged 5–12 years, referred to outpatient rehabilitation clinics. Participant recruitment focused on maximizing ecological validity by engaging families actively seeking services, ensuring the sample reflected real-world clinical presentations. A targeted sample of 60 child-parent dyads was recruited to provide robust data for the planned analyses. This sample size is well-supported for correlational and regression analyses, providing adequate power to detect the strong effects hypothesized in this clinical population.

### Procedure and measures

Eligibility was determined through a rigorous, multi-method diagnostic process to ensure sample homogeneity. First, a comprehensive clinical diagnosis of ASD was confirmed by a licensed clinic psychiatrist using DSM-5 criteria. Second, to precisely operationalize the “low support needs” profile, standardized criteria were applied: a full-scale IQ > 80 (Verified by clinic-administered assessments) and scores at or above validated clinical thresholds on the Autism Spectrum Screening Questionnaire (ASSQ) from both parents and teachers. This dual-informant approach on the ASSQ strengthens diagnostic confidence by incorporating perspectives from both home and school settings. The primary study measures of sensory processing, social skills, and caregiver burden were then completed by the participating parent. The parent report is the established and ecologically valid method for assessing these constructs, as parents have unique, longitudinal insight into their child’s everyday behaviors and the family’s subjective caregiving experience, which is the central focus of this study.

### Inclusion and exclusion criteria

Inclusion criteria were: (1) a formal DSM-5 diagnosis of ASD by a clinic psychiatrist, (2) age between 5 and 12 years, (3) a documented IQ > 80, (4) scoring ≥19 on the parent ASSQ and ≥22 on the teacher ASSQ (22), (5) willingness of both the child and their parent to provide informed assent/consent. Exclusion criteria included: (1) comorbid neurological or genetic syndromes known to affect sensory or social functioning (e.g., cerebral palsy, Fragile X syndrome), (2) significant uncorrected sensory impairments (blindness, deafness), or (3) major uncontrolled medical conditions. Following ethical approval and informed consent, parents completed the study questionnaires. Data were analyzed using SPSS-26. Descriptive statistics summarized the sample. Inferential analyses included Pearson’s correlation and stepwise multiple regression to examine relationships and identify predictors of parental care burden. All procedures adhered to ethical standards regarding confidentiality and voluntary participation.

### Instruments

#### Demographic information checklist

This is a researcher-made checklist. After providing necessary explanations about the research and its objectives, it poses questions designed to identify demographic factors. It includes items such as age, gender, educational level, parental education, family history of illness, and socio-economic status.

#### Autism Spectrum Screening Questionnaire

The Autism Spectrum Screening Questionnaire (ASSQ) is a 27-item screening instrument developed by Ehlers et al. (22). Each item is rated on a 3-point scale (0 = “not true,” 1 = “somewhat true,” 2 = “certainly true”) by parents or teachers. The total score provides an indicator of traits associated with Autism Spectrum Disorder (ASD). In this study, we employed the ASSQ as a secondary verification tool to support the clinical diagnosis of ASD and to characterize the sample. The clinical diagnosis was established by a qualified clinic psychiatrist based on a comprehensive assessment and DSM-5 criteria. To align our sample with the operational definition of autistic children with low support needs, we utilized established ASSQ cut-off scores as one of the inclusion criteria. A child was included if they met the clinical diagnosis and scored at or above the recommended screening threshold: a score of ≥19 on the parent-completed ASSQ or a score of ≥22 on the teacher-completed ASSQ (22). These specific cut-offs were selected because they have been validated in prior research for identifying children with ASD who exhibit subtler presentations, often without co-occurring intellectual disability, which corresponds to our target population. This dual-informant (parent and teacher) and multi-method (clinical diagnosis plus screening score) approach was implemented to enhance the phenotypic specificity of our sample. For the current sample, we calculated the internal consistency of the ASSQ (23). In the present study, the calculated Cronbach’s alpha was 0.76 for parent reports and 0.81 for teacher reports, indicating acceptable internal consistency within our population.

#### Caregiver burden inventory

Parental caregiving burden was assessed using the Zarit Burden Interview (24). This 22-item scale measures subjective burden across domains such as personal strain, social strain, emotional strain, and economic strain, with responses ranging from 0 (Never) to 4 (Nearly Always). The original version reports excellent reliability (test-retest r=0.71, Cronbach’s α = 0.91) (24). For the current sample, we assessed the scale’s internal consistency, which yielded a Cronbach’s alpha of 0/89, confirming its reliability within our study population.

#### Sensory profile - school companion

Sensory processing patterns were evaluated using the Sensory Profile School Companion (25), a standardized 62-item parent-report questionnaire for children aged 3–11 years. It measures four quadrants: Sensory Registration, Sensation Seeking, Sensory Sensitivity, and Sensation Avoiding. The original manual reports high internal consistency (α = 0.83-0.95) and test-retest reliability (r = 0.80-0.95). To ensure the tool’s appropriateness for our sample, we computed its internal consistency (25). The Cronbach’s alpha values for the four quadrants in this study were as follows: Sensory Registration α = 0.90, Sensation Seeking α = 0.88, Sensory Sensitivity α = 0.86, and Sensation Avoiding α = 0.87. These results indicate strong internal consistency within our participant group.

#### Gresham and Elliott social skills rating scale - parent version

Developed by (26), it has three versions: for parents, teachers, and students. This study used the Parent Version, which has 52 items and measures two factors: (a) the Social Skills factor, which includes the subscales of Cooperation, Assertion, and Responsibility; and (b) the Problem Behaviors factor, which includes the subscales of Externalizing Behaviors, Internalizing Behaviors, and Hyperactivity (27). In the present study, the initial section on social skills was utilized, which includes subscales such as Cooperative Behaviors (interacting with others, helping others, sharing materials, following rules), Assertive Behaviors (initiating behaviors, introducing oneself and inquiring about others, responding appropriately to others’ actions), Self-Control (behaving appropriately in difficult situations such as being teased, appropriate behavior in challenging situations like waiting for a turn). The sum of scores in these subscales constituted the total social skills score. For our sample, we calculated the internal consistency for both factors. The Cronbach’s alpha was 0.91 for the Social Skills factor and 0.87 for the Problem Behaviors factor, confirming good reliability in the present context.

## Results

The demographic information of the study sample is reported in **Table 1** and indicates that the total sample consisted of 60 children with ASD, of whom 36 (60%) were boys and 24 (40%) were girls. The largest age group consisted of 24 children (40%) in the 7–9 year range. Twenty children (33.3%) were in the 5–6 year age range, and 16 children (26.7%) were in the 10–12 year age range. The mean age for the entire sample was 7.92 years with a standard deviation of 2.17. Regarding educational level, 20 children (33.3%) were in preschool, 24 children (40%) were in grades 1 to 3 of elementary school, and 16 children (26.7%) were in grades 4 to 6.

**Table 1:** Descriptive statistics of the research variables (N = 60).

Variable	Mean	SD	Min	Max
Parental Care Burden				
Personal Strain	11.57	7.07	3	22
Social Strain	10.87	7.37	2	22
Emotional Strain	11.38	6.96	3	22
Economic Strain	10.27	7.31	4	21
Total Care Burden	35.22	23.80	10	80
Social Skills				
Social Skill	38.38	17.90	15	66
Problem Behavior	12.72	7.37	3	24
Sensory Processing				
Sensory Registration	37.28	18.19	17	70
Sensation Seeking	23.13	13.41	9	47
Sensory Sensitivity	20.58	10.98	7	35
Sensory Avoiding	32.80	15.46	14	66

The socio-economic status of the sample was as follows: 18 children (30%) were from families with a low socio-economic status, 31 children (51.7%) from families with a medium status, and 11 children (18.3%) from families with a high status.

Regarding birth order, the highest number was for only children, with 35 children (58.3%), followed by first-born children with 14 (23.3%), and finally second-born children with 11 (18.4%). Forty-seven children (78.3%) had no family history of autism spectrum disorder, while 13 children (21.7%) had a positive family history.

Concerning the education level of the parents of the children with autism, the highest frequency was 29 parents (48.3%) with a diploma or associate’s degree. This was followed by 20 parents (33.3%) with a bachelor’s degree or higher, 6 parents (10%) with a master’s degree or higher, and finally 5 parents (8.3%) with education below high school diploma.

As reported in the **Table 1**, the age of the mothers in the sample ranged from 28 to 45 years, with a mean and standard deviation of 36.30 and 4.57 years, respectively. The most frequent age range for mothers was 36–40 years, with 28 individuals.

Finally, the reported intelligence quotient (IQ) of the sample showed a mean IQ of 89.80 with a standard deviation of 3.013. The IQ range was between 80 and 98.

In the subsequent section and **Table 1**, a summary of the statistical indices of the participants’ scores for each variable is provided.

Subsequently, Pearson correlation method was used to examine and explain the relationships between the research variables; the results are presented in **Table 2**.

**Table 2:** Correlations between sensory processing patterns and parental care burden subscales.

Variable	Personal strain	Social strain	Emotional strain	Economic strain	Total care burden
Sensory Registration	0.46*	0.44*	0.48*	0.42*	0.52*
Sensation Seeking	0.50*	0.52*	0.53*	0.48*	0.56*
Sensory Sensitivity	0.48*	0.47*	0.51*	0.45*	0.54*
Sensory Avoiding	0.51*	0.53*	0.54*	0.49*	0.58*

*p < 0.001. All correlations are positive.

### Correlations between sensory processing patterns and parental care burden

Bivariate correlations between sensory processing patterns and the subscales of parental care burden were examined (**Table 2**). All four sensory patterns showed significant positive correlations with the total care burden score and its subscales. The strength of these correlations ranged from moderate to strong (0.42 to 0.58), with Sensory Avoiding and Sensation Seeking showing the strongest overall associations with burden.

### Correlations between social skills and parental care burden

Correlations between social skills, problem behavior, and care burden are presented in **Table 3**. As hypothesized, the Social Skill score was significantly negatively correlated with all burden subscales and the total score (-0.48 to -0.59). Conversely, Problem Behavior was significantly positively correlated with all burden measures (0.46 to 0.55).

**Table 3:** Correlations between Social Skills, Problem Behavior, and Parental Care Burden.

Variable	Personal strain	Social strain	Emotional strain	Economic strain	Total care burden	Social skill
Social Skill	-0.52*	-0.51*	-0.55*	-0.48*	-0.59*	—
Problem Behavior	0.52*	0.50*	0.53*	0.46*	0.55*	-0.58*

*p < 0.001.

### Regression analyses predicting parental care burden

Two sets of regression analyses were conducted to examine the unique predictive value of sensory patterns and social skills.

1. Regression with Sensory Processing Patterns: A standard multiple regression was performed with the four sensory patterns as simultaneous predictors of total care burden. The model was significant, F(4, 55) = 9.42, p < 0.001, explaining 40.7% of the variance (Adj. R² = 0.38). As shown in **Table 4**, Sensory Avoiding (β = 0.31, p = 0.02) and Sensory Sensitivity (β = -0.28, p = 0.03) emerged as significant unique predictors.
2. Regression with Social Skills and Problem Behavior: A hierarchical regression was conducted. In Step 1, Social Skill alone was entered, significantly predicting 34.8% of the variance in care burden (F(1,58)=30.95, p<0.001, β = -0.59). In Step 2, Problem Behavior was added. The overall model remained significant (F(2,57)=20.11, p<0.001) and the explained variance increased to 41.4% (ΔR² = 0.066, p = 0.02). In the final model (**Table 4**), both Social Skill (β=-0.42, p=0.003) and Problem Behavior (β=0.28, p=0.02) were significant unique predictors.

**Table 4:** Summary of regression analyses for predicting total parental care burden.

Model & predictors	B (SE)	β	t	p	95% CI for B
Model A: Sensory Patterns					
Constant	2.15 (3.88)		0.55	0.58	[-5.62, 9.92]
Sensory Registration	0.15 (0.15)	0.11	0.97	0.34	[-0.16, 0.45]
Sensation Seeking	0.25 (0.20)	0.18	1.28	0.21	[-0.14, 0.64]
Sensory Sensitivity	0.62 (0.27)	0.28	2.27	0.03	[0.08, 1.16]
Sensory Avoiding	0.51 (0.21)	0.31	2.42	0.02	[0.09, 0.93]
Model B: Social/Behavioral Factors (Final Model)					
Constant	58.22 (5.10)		11.42	<0.001	[48.00, 68.44]
Social Skill	-0.59 (0.19)	-0.42	-3.10	0.003	[-0.97, -0.21]
Problem Behavior	0.90 (0.37)	0.28	2.44	0.02	[0.16, 1.64]

Model A: R² = 0.407, Adj. R² = 0.38, F(4,55)=9.42, p<0.001. Model B: R² = 0.414, Adj. R² = 0.39, F(2,57)=20.11, p<0.001. B = unstandardized coefficient; SE = standard error; β = standardized coefficient.

The correlational analyses confirm significant relationships in the expected directions. Regression analyses indicate that both sensory processing patterns (particularly Sensory Sensitivity and Avoiding) and social/behavioral factors (Social Skills and Problem Behavior) are significant and unique predictors of parental care burden. Together, these sets of variables explain a substantial and statistically reliable portion of the variance in caregiver burden (Approximately 41%).

## Discussion

The present study investigated the predictive role of sensory processing patterns and social skills in parental care burden among parents of autistic children with low support needs. The findings revealed significant, moderate-to-strong correlations and regression models explaining a substantial portion of the variance in caregiver burden, providing clear support for the study’s hypotheses.

The results demonstrated significant positive correlations between all four sensory processing patterns (Sensory Registration, Sensation Seeking, Sensory Sensitivity, and Sensory Avoiding) and the total parental care burden score, as well as its individual subscales (personal, social, emotional, and economic strain). These correlations ranged from 0.42 to 0.58, indicating that greater sensory processing difficulties are associated with a higher reported burden. This aligns with previous research highlighting the stressogenic nature of sensory dysregulation in ASD (28). For instance, a child’s sensory sensitivity leading to avoidance of crowded environments or tactile defensiveness disrupting daily care routines can create constant, low-grade stressors that accumulate into a significant caregiving load (29).

Furthermore, the regression analysis clarified the unique predictive contribution of these patterns. When considered together, the four sensory patterns explained a significant 40.70% of the variance in care burden. Notably, Sensory Sensitivity (β = 0.28) and Sensory Avoiding (β = 0.31) emerged as significant unique predictors. This suggests that specific aspects of sensory processing—particularly a child’s heightened reactivity to stimuli (Sensitivity) and their active efforts to withdraw from stimuli (Avoidance)—are most directly taxing for parents. These behaviors can be unpredictable and challenging to manage in everyday life, demanding high levels of parental vigilance and adaptation, thereby increasing the subjective burden (30). The finding that Sensory Registration and Sensation Seeking did not contribute unique predictive variance in the combined model does not negate their significant bivariate relationships; rather, it indicates their shared influence is captured by the other two patterns in a multivariate context.

As hypothesized, a significant negative correlation was found between children’s social skills and parental care burden (r = -0.59), while problem behavior was positively correlated with burden (r = 0.55). This pattern held across all burden subscales. These results confirm that deficits in core social competencies, such as cooperation, assertion, and self-control, are closely linked to increased caregiver strain. Conversely, the presence of externalizing or internalizing problem behaviors directly adds to the parenting challenge (31).

The hierarchical regression analysis provided deeper insight. Social skills alone accounted for 34.8% of the variance in burden. When problem behavior was added to the model, the total explained variance increased to 41.4%, with both Social Skill (β = -0.42) and Problem Behavior (β = 0.28) remaining significant unique predictors. This indicates that while social skills and problem behavior are related (r = -0.58), each contributes independently to parents’ experience of burden. A child may have limited social skills, leading to difficulties in family and community interactions, which is burdensome. Independently, the same child may exhibit problem behaviors like hyperactivity or emotional outbursts, which create an additional layer of stress (32). This dual pathway underscores the complex challenges parents face and suggests that interventions targeting both social competency and behavioral regulation may be most effective in alleviating burden (11).

The findings can be interpreted through the lens of the Underconnectivity Theory, which posits neural integration deficits in autism. These deficits may manifest as the sensory processing irregularities and social skill impairments observed here (18). The child’s resulting behaviors—whether sensory-driven avoidance or socially awkward interactions—create a caregiving environment characterized by high demand and low predictability, directly fueling parental stress (33). This creates a potential vicious cycle: parental stress may reduce the capacity for sensitive, responsive parenting, which could in turn exacerbate the child’s regulatory difficulties (34).

Practically, these results strongly advocate for a holistic assessment and intervention approach. Clinicians should routinely screen for sensory processing challenges and social skill deficits, as these are not merely core features of ASD but are significant contributors to family distress. Parent-focused interventions, such as psychoeducation about sensory processing, training in behavior management techniques, and social skills facilitation strategies, are crucial. Empowering parents to understand and effectively respond to their child’s sensory and social needs can break the stress cycle, improving both child outcomes and parental well-being (17).

This study has several limitations. First, the use of a convenience sample from clinical settings limits the generalizability of the findings to the broader population of autistic children and their families. Second, the reliance on parent-reported measures for all constructs, while valid for assessing subjective burden, introduces the possibility of common method variance. Future research would benefit from multi-method assessments, including direct child observation, teacher reports, and physiological measures of stress. Third, the cross-sectional design precludes causal inference. Longitudinal studies are needed to examine the directional and potentially reciprocal relationships between child characteristics and parental burden over time. Finally, the sample size, though adequate for the analyses performed, was moderate. Future studies with larger, more diverse samples could employ more complex models to examine mediating (e.g., parental self-efficacy) and moderating (e.g., social support) variables.

Despite these limitations, the study makes a clear contribution by quantifying the substantial shared variance between sensory-social profiles of autistic children with low support needs and their parents’ care burden. It underscores the necessity of moving beyond a child-centric view of intervention to embrace a family-system perspective that actively addresses parental burden as a key outcome.

## Conclusion

This descriptive-correlational study examined the relationships between sensory processing patterns, social skills, and parental care burden in a sample of autistic children with low support needs aged 5–12 years. The findings robustly demonstrate that both domains are significantly and substantially linked to the caregiving experience.

The results confirmed that heightened sensory processing difficulties, particularly in the domains of Sensory Sensitivity and Sensory Avoiding, are significant predictors of increased parental burden. Concurrently, lower levels of Social Skills and higher levels of Problem Behavior in children independently and collectively predicted greater caregiver strain. Together, sensory and social-behavioral factors explained a considerable portion (approximately 41%) of the variance in parental burden, underscoring their clinical relevance.

These findings translate into a clear imperative for family-centered practice. They highlight that the core and associated features of autism—sensory dysregulation and social-communication deficits—are not solely challenges for the child but are primary drivers of family stress. Consequently, comprehensive assessment and intervention must extend beyond the child to directly address parental burden. Evidence-based interventions that equip parents with strategies to manage sensory-related behaviors, foster social skill development, and address problem behaviors are essential. By adopting this dual focus on child support and parent empowerment, clinical practice can more effectively alleviate caregiver burden, enhance family quality of life, and disrupt the potential cycle of stress that impedes positive outcomes for autistic children. Future research employing longitudinal and multi-method designs is needed to further elucidate these pathways and evaluate targeted family support interventions.

## Data Availability

The original contributions presented in the study are included in the article/supplementary material. Further inquiries can be directed to the corresponding author.
